# Changes in Vitamin A Levels and the Effect of Early Vitamin A Supplementation on Vitamin A Levels in Infants Throughout the First 6 Months of Life: A Prospective Cohort Study in Chongqing, China

**DOI:** 10.3389/fpubh.2021.650823

**Published:** 2021-04-27

**Authors:** Huan Liu, Qixiong Chen, Linchao Yu, Ting Yang, Jie Chen, Jingkun Miao, Tingyu Li

**Affiliations:** ^1^Chongqing Key Laboratory of Child Nutrition and Health, Ministry of Education Key Laboratory of Child Development and Disorders, Children's Nutrition Research Center, National Clinical Research Center for Child Health and Disorder, Children's Hospital of Chongqing Medical University, Chongqing, China; ^2^Department of Pediatrics, Chongqing Traditional Chinese Medicine Hospital, Chongqing, China; ^3^Neonatal Screening Center, Chongqing Health Center for Women and Children, Chongqing, China

**Keywords:** vitamin A, vitamin A deficiency, neonate, infant, supplements

## Abstract

**Objectives:** This study aimed to explore the changes in infant vitamin A (VA) status and the effect of early VA supplementation on VA level throughout the first 6 months of life.

**Methods:** A prospective cohort study was conducted in Chongqing, China. A total of 1,016 healthy infants were enrolled at birth. Then, 930, 882, 854 and 822 healthy infants were followed up at postnatal day 7 and postnatal months 1, 3, and 6, respectively. Blood samples and dietary survey and physical development data were collected. Serum VA was measured by chromatography tandem-mass spectrometry and was classified according to the VA deficiency (VAD) criteria for older children aged 6–70 months (<0.70, 0.70–1.05, ≥1.05 μmol/L). Normally distributed continuous variables are presented as the mean ± standard deviation. The categorical variables are described by the frequency and percentage (%). The reference interval for the VA level was the 2.5th−97.5th percentile. Changes in VA status with age and the relationship of VA supplementation with VA level were investigated by generalized estimating equations followed by Bonferroni *post hoc* test, controlling for the effects of feeding pattern and sex.

**Results:** Infant VA levels increased significantly from 0.499 ± 0.146 to 1.061 ± 0.414 μmol/L with age at 6 months, even without VA supplementation (*P* < 0.05). From birth to 6 months, the percentage of infants with a VA level <0.70 μmol/L decreased from 88.6 to 19.5%. During follow-up, no infant demonstrated clinical VAD conditions, such as night blindness, conjunctival xerosis or Bitot's spots. Less than 7.0% of infants were underdeveloped in terms of weight, length and head circumference. The VA status of infants with VA≥0.588 μmol/L at birth gradually increased to adequate VA (VA ≥ 1.05 μmol/L) at 6 months. For these infants, there was no significant difference in VA level between the VA supplementation and non-supplementation groups (*P* > 0.05). Infants with VA <0.430 μmol/L at birth still had VA <0.70 μmol/L at 6 months; in this group, VA levels increased by 0.08 μmol/L more among supplemented infants than among non-supplemented infants (*P* < 0.05).

**Conclusions:** A low VA level among neonates at birth may be a normal physiological state and may increase with age; thus, not all neonates may need early VA supplementation. More multicenter studies are needed to determine a new cutoff point for the diagnosis of neonatal VAD and the administration of nutritional intervention.

## Introduction

Vitamin A (VA) is an essential fat-soluble micronutrient for normal functioning of the visual system as well as for immune response, gene expression, reproduction, embryogenesis, hematopoiesis, normal growth and neurocognitive development (1–4). VA nutrition is especially important in the first 1,000 days of life. VA deficiency (VAD) in early life can lead to numerous physiological dysfunctions and weaken host resistance to infection (5–7). Worse yet, VAD might even significantly increase infant morbidity and mortality (8–11).

According to the World Health Organization (WHO) (6), a serum VA level <0.70 μmol/L is the diagnostic criterion for VAD in adults and older children aged 6–70 months. In most studies, this diagnostic criterion was also used in neonates. At present, as living standards have improved, the VAD rates of adults and older children have fallen significantly worldwide (12). However, the percentage of neonates with VAD remains as high as 42–82% according to some studies (13–16). In a previous study, we also found a high incidence of neonatal VAD (60.1%) in areas where VAD was uncommon (17, 18). Is VAD in newborns truly that prevalent? Other studies have suggested that the biological levels of neonatal VA at birth may be much lower than the level of VA in adults (13–15), speculating that the application of existing VAD diagnostic criteria for older children may overestimate the rate of VAD in newborns (16). However, this hypothesis was not validated in that study.

In addition, there are no clear clinical guidelines on whether neonates should be administered VA supplements in the early postpartum period (1, 19). On the one hand, if the incidence of VAD among neonates is not truly as high as has been reported, there is a risk of VA overdose poisoning with supplementation (20). On the other hand, findings on the effect of neonatal VA supplementation on VA status have been inconclusive (21, 22).

Therefore, we first followed up the VA nutritional status among healthy infants throughout the first 6 months of life by a large prospective longitudinal study to prove that the low VA level of neonates at birth may be a normal physiological state and that the percentage of neonates with VAD may be overestimated. Moreover, we explored the influence of postnatal VA supplementation on VA levels throughout the first 6 months of life to provide solid evidence regarding whether early VA intervention is necessary for infants.

## Materials and Methods

### Study Population and Design

A population-based, prospective longitudinal study following infants throughout the first 6 months of life was conducted between May 2018 and May 2019 in Chongqing, China. This study was carried out in two representative large tertiary grade A hospitals in Chongqing, the Second Affiliated Hospital of Chongqing Medical University, located in an urban area, and Qianjiang Central Hospital, located in a suburban area. Chongqing is located in southwest China and is one of the most important central cities in China, with a total area of 82,400 square kilometers, a permanent resident population of 31,243,200 and a birth population of 326,200 per year.

All infants born in these two hospitals from May 2018 to May 2019 who met the following inclusion criteria were enrolled in this study from birth: (1) parents who agreed to participate in this study; (2) gestational age ≥37 weeks and <42 weeks; (3) infant birth weight ≥2,500 g and ≤ 4,000 g; (4) singleton pregnancy; (5) prenatal examination showing no abnormalities; and (6) no metabolic or infectious diseases at birth. The exclusion criteria for the infants were as follows: (1) family disapproval of participation and (2) hospitalization for infectious diseases after birth.

**Figure 1:**
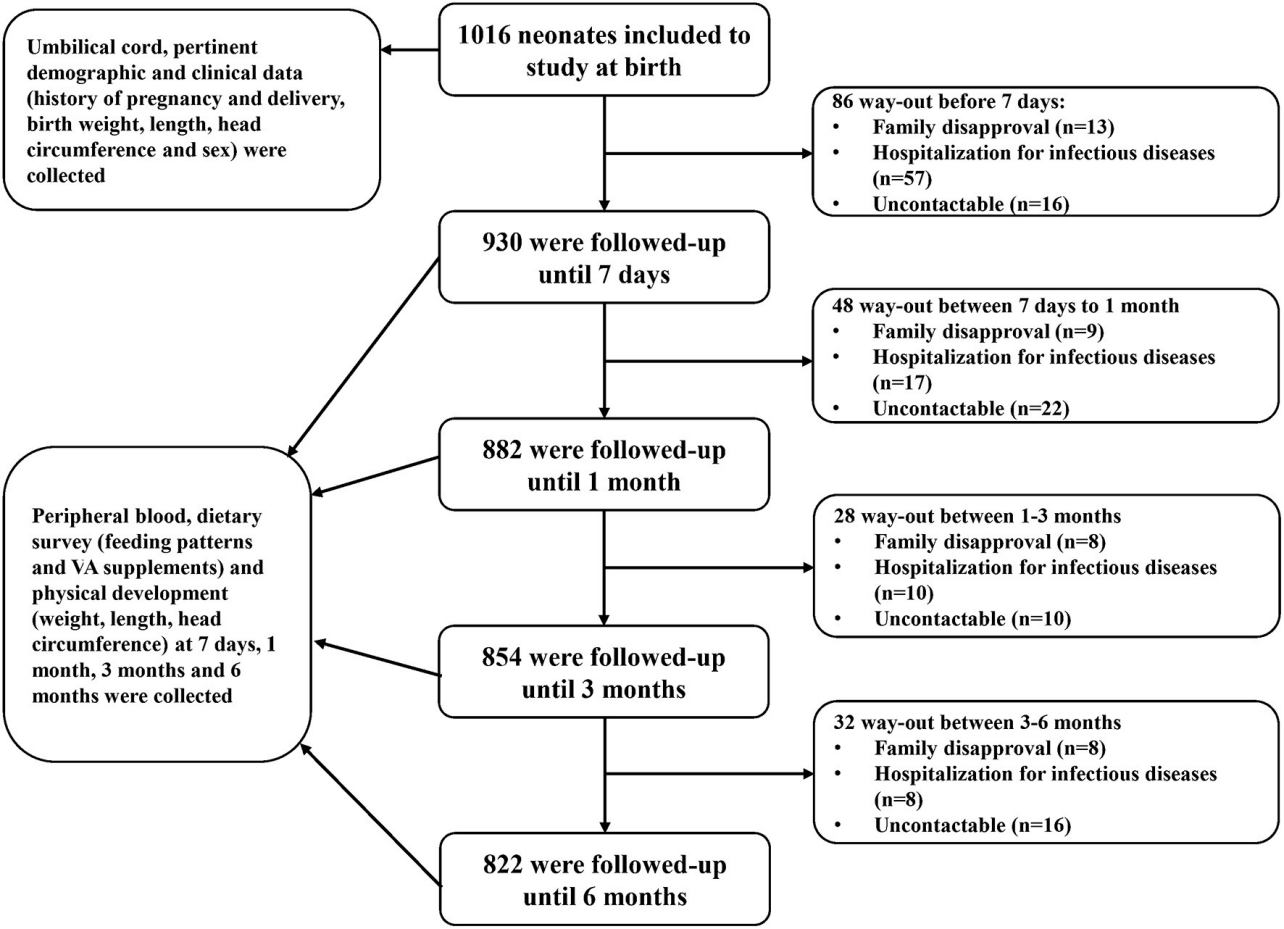
Flowchart diagram of follow-up. Infants were enrolled from birth and were followed up at postnatal day 7 and postnatal months 1, 3, and 6. At birth, blood samples and pertinent demographic and clinical data were collected. During follow-up, blood samples and dietary survey and physical development data were collected.

During the above-stated period, a total of 1,827 neonates were born in the two hospitals, and 1,016 neonates met the inclusion criteria and were included at birth in this study. The sample size calculation formula used was (23) *n* = $\frac{p\left(1-p\right)}{e^{2}}$, in which *p*, the estimated prevalence, was 18% (23) and *e*, the standard error of the estimate, was 2% (23). The required sample size was initially calculated as 369. The 20% increase in sample size accounting for the sampling error (23) resulted in a total of 443. Allowing for 20% lost to follow-up (24), the final required sample was 554. The number of samples included in this longitudinal study was 1,016, well-over the calculated 554. Then, 930, 882, 854, and 822 infants were followed up at postnatal day 7 and postnatal months 1, 3, and 6, respectively (Figure 1). Across the 6-month follow-up, the parents of 64 (6.3%) infants could not be contacted and were lost to follow-up, the parents of 38 (3.7%) withdrew their consent, and 92 (9.1%) had been hospitalized for infectious diseases. Ultimately, 822 (80.9%) infants completed the study. The characteristics (sex, gestational age, weight, length, and head circumference at birth) of the infants who completed the 6-month follow-up were similar to those of the infants who did not (data not shown). Our analysis was based on data from 1,016, 930, 882, 854, and 822 infants at birth, postnatal day 7, and postnatal months 1, 3, and 6, respectively.

The maternal VA level of the infants included in this study was (1.056 ± 0.468) μmol/L, and the maternal VAD rate was 23.3%. The intake of maternal dietary VA was (1,033.2 ± 599.9) μg RAE/d, and the insufficiency rate of dietary intake was 27.6% (see Supplementary Table 1).

All subjects gave their informed consent for inclusion before they participated in the study. The study was conducted in accordance with the Declaration of Helsinki, and the protocol was approved by the Medical Ethics Committee of Children's Hospital Affiliated with Chongqing Medical University (022/2014).

### Study Procedures

#### Clinical Data Collection

After obtaining informed consent, the history of pregnancy and delivery, gestational age, and sex of the participants enrolled at birth were collected from the electronic medical records of the participants. Infant physical development was monitored by measuring weight, length and head circumference at birth and at postnatal day 7 and postnatal months 1, 3, and 6. Weight was measured to the nearest 10 g with an electronic baby scale (LEKA, HW-B60). Length was measured using a measuring bed (LEKA, HW-B60), and head circumference was measured using a flexible ruler (Deli, 8213), both with a precision of 1 mm. The measured value was the average value after three repeated measurements.

#### Dietary Data Collection

Infant dietary information, including feeding patterns and VA supplementation, was collected using a questionnaire. At the 7-day, 1-month, 3-month, and 6-month scheduled visits, the diets of the infants since their last visits were reviewed and recorded. Feeding patterns were categorized into three groups (breast feeding, mixed feeding and artificial feeding). According to the infant nutrition and health guidelines (25), infants under 6 months of age are recommended to receive 400IU of vitamin D (VD) supplementation daily. Currently on the market, the AD mixture containing both VA and VD is widely used for VD supplementation in infants (one drop daily, each drop contains 450 μg RAE/d of VA and 500 IU of VD). Therefore, although there are no clear guidelines on VA supplementation for little infants (1, 19), some healthy infants in China can receive VA supplementation along with VD supplementation from the AD Mixture. The infants were divided into a VA supplementation group (those who took VA supplements at least one drop per week after birth, 450 μg RAE of VA per drop) and a non-supplementation group (those who took VA supplements <1 drop per week, 450 μg RAE of VA per drop).

#### Blood Sample Collection

Samples of cord blood at birth and capillary blood at 7 days, 1 month, 3 months, and 6 months postnatally were collected. Blood samples (0.1 ml) were collected, centrifuged, and stored at −80° within 12 h. The retinol concentration was detected within 2 months.

#### Measurement of Serum Retinol

The measurement of retinol level in serum is a common way to assess the level of VA in the body. The samples were analyzed at the Pediatric Research Institute, Children's Hospital Affiliated with Chongqing Medical University. The retinol concentration in serum was measured by high-performance liquid chromatography tandem-mass spectrometry (HPLC-MS/MS) using an API3200 (AB SCIEX, 500 Old Connecticut Path Framingham, MA, USA). Briefly, serum samples (20 μl) were deproteinized with methanol containing an internal standard (0.5 μg/ml d6-retinyl acetate), extracted with hexane, evaporated to dryness under nitrogen, and reconstituted in methanol. The retinol in serum was separated by HPLC on a Shimadzu C18 75 ×2.0 mm column and quantitated by MS. All procedures were performed in a dark room to protect the samples from light. The lowest sensitivity of the measurements was 0.014 μmol/L for retinol.

#### Definitions

Infants with weight, length or head circumference below the third percentile were considered underweight, stunted or underdeveloped for head circumference, respectively. According to the Growth Standards and Growth Charts for Chinese Children (26), the third percentiles of weight for boys at birth, 1 month, 3 months, and 6 months are 2,620.0, 3,580.0, 5,370.0, and 6,800.0 g, respectively; the third percentiles of weight for girls are 2,570.0, 3,380.0, 4,960.0, and 6,340.0 g, respectively. The third percentiles of length for boys are 47.1, 51.0, 57.7, and 64.0 cm, respectively; the third percentiles of length for girls are 46.6, 50.0, 56.5, and 62.5 cm, respectively. The third percentiles of head circumference for boys are 32.3, 34.6, 38.1, and 41.2 cm, respectively; the third percentiles of head circumference for girls are 31.8, 33.9, 37.2, and 40.2 cm, respectively. According to the recommendation of the WHO (6, 15), the VA status in children aged 6–70 months is classified according to the following criteria: VAD, VA <0.70 μmol/L; marginal VAD, VA 0.70–1.05 μmol/L; and adequate VA, VA ≥1.05 μmol/L. Since there are no specific VA level grouping criteria for infants under 6 months of age, the above criteria were used in this study to group the VA statuses of the study population in the initial analysis. Clinical VAD, evidenced by conditions including night blindness, conjunctival xerosis, Bitot's spots, corneal xerosis, corneal ulceration/keratomalacia affecting <1/3 of the corneal surface or ≥1/3 of the corneal surface, or corneal scarring (14), was evaluated by specialists.

### Statistical Analysis

Statistical analysis was performed using the SPSS software system, version 20.0. Figures were drawn using the GraphPad Prism software system, version 5.0. Normally distributed continuous variables are presented as the mean ± standard deviation (SD) (2.5th−97.5th percentile). Categorical variables are described by the frequency and percentage (%). The reference interval for the VA level was the 2.5th−97.5th percentile. Changes in VA level and distribution with age were determined with generalized estimating equations. These equations were also used to investigate the relationship between VA supplementation and serum VA levels with age, controlling for the effects of feeding pattern (Breast feeding vs. Mixed feeding vs. Artificial feeding) and sex (Female vs. Male). The Bonferroni method was used to correct for multiple comparisons following the use of the generalized estimating equations. Statistical significance was set at *P* < 0.05.

## Results

### Infant Characteristics

The mean gestational age at birth was 39.5 ± 1.1 weeks (Table 1). The ratio of males to females at birth was ~1.2:1 (553:463). The average weight was 3,321.9 ± 319.4 g, the average length was 49.7 ± 1.5 cm and the average head circumference was 34.1 ± 1.3 cm at birth. During the 6-month follow-up, 0.0–3.9% of infants were underweight, 2.7–6.8% of infants were stunted, and 0.2–5.8% were underdeveloped for head circumference. The majority of infants (72.6–72.9%) were exclusively breastfed, and <7% were fed artificially. More than half of the infants (52.7–53.1%) took VA supplements.

**Table 1:** Clinical characteristics and VA status of infants during the first 6 months of life in Chongqing, Southwest China.

	**At birth**		**PN-7d**		**PN-1m**		**PN-3m**		**PN-6m**	
	**n**	Mean ± SD^*^ or percentage	***n***	Mean ± SD ^*^ or Percentage	**n**	Mean ± SD ^*^ or percentage	***n***	Mean ± SD^*^ or percentage	***n***	Mean ± SD^*^ or percentage
Gestational age (weeks)	1,016	39.5 ± 1.1 (37.1–41.4)	−	−	−	−	−	−	−	−
Sex										
Male	553	54.4%	502	54.0%	468	53.1%	452	52.9%	437	53.2%
Female	463	45.6%	428	46.0%	414	46.9%	402	47.1%	385	46.8%
Weight (g)	1,016	3,321.9 ± 319.4 (2,677.5–3,900.0)	930	3,518.8 ± 335.4(2,855.0–4,115.0)	882	4,600.1 ± 381.5(3,796.1–5,310.0)	854	6,581.9 ± 557.9(5,450.7–7,665.3)	822	7,869.6 ± 688.6(6,449.1–9,201.9)
< P3^#^	11	1.1%	−	−	0	0.0%	9	1.1%	32	3.9%
Length (cm)	1,016	49.7 ± 1.5(45.7–52.7)	930	50.9 ± 1.5(47.0–54.0)	882	54.6 ± 1.8(50.4–57.8)	854	61.1 ± 2.3(56.2–65.5)	822	67.6 ± 2.5(62.3–72.5)
< P3	69	6.8%	−	−	24	2.7%	41	4.8%	43	5.2%
Head circumference (cm)	1,016	34.1 ± 1.3(31.7–36.4)	930	34.9 ± 1.3(32.7–37.2)	882	37.9 ± 1.4(35.3–40.8)	854	41 ± 1.6(38.0–44.1)	822	42.8 ± 1.6(39.7–46.0)
< P3	43	4.2%	−	−	2	0.2%	16	1.9%	48	5.8%
**Feeding pattern**										
Breast feeding	−	−	676	72.7%	641	72.7%	620	72.6%	599	72.9%
Mixed feeding	−	−	198	21.3%	185	21.0%	181	21.2%	174	21.2%
Artificial feeding	−	−	56	6.0%	56	6.3%	53	6.2%	49	5.9%
**VA supplement**										
No	−	−	436	46.9%	416	47.2%	403	47.2%	389	47.3%
Yes	−	−	494	53.1%	466	52.8%	451	52.8%	433	52.7%
**VA level** ^**#**^	1,016	0.499 ± 0.146 (0.277–0.829)	930	0.508 ± 0.165 (0.249–0.893)	882	0.678 ± 0.325 (0.219–1.474)	854	0.808 ± 0.314 (0.311–1.551)	822	1.061 ± 0.414 (0.494–2.042)
<0.70	900	88.6%	819	88.1%	522	59.2%	352	41.2%	160	19.5%
0.70-1.05	114	11.2%	103	11.1%	230	26.1%	326	38.2%	327	39.8%
≥1.05	2	0.2%	8	0.8%	130	14.7%	176	20.6%	335	40.7%

* *meaning the 2.5th−97.5th percentile*.

# *The VA level is measured in μmol/L*.

*VA, vitamin A; PN-7d, postnatal day 7; PN-1m, postnatal month 1; PN-3m, postnatal month 3; PN-6m, postnatal month 6; P3, the third percentile*.

*^#^ < P3 means physical underdevelopment for weight, length or circumference*.

### Changes in Infant VA Status Throughout the First 6 Months of Life

**Figure 2:**
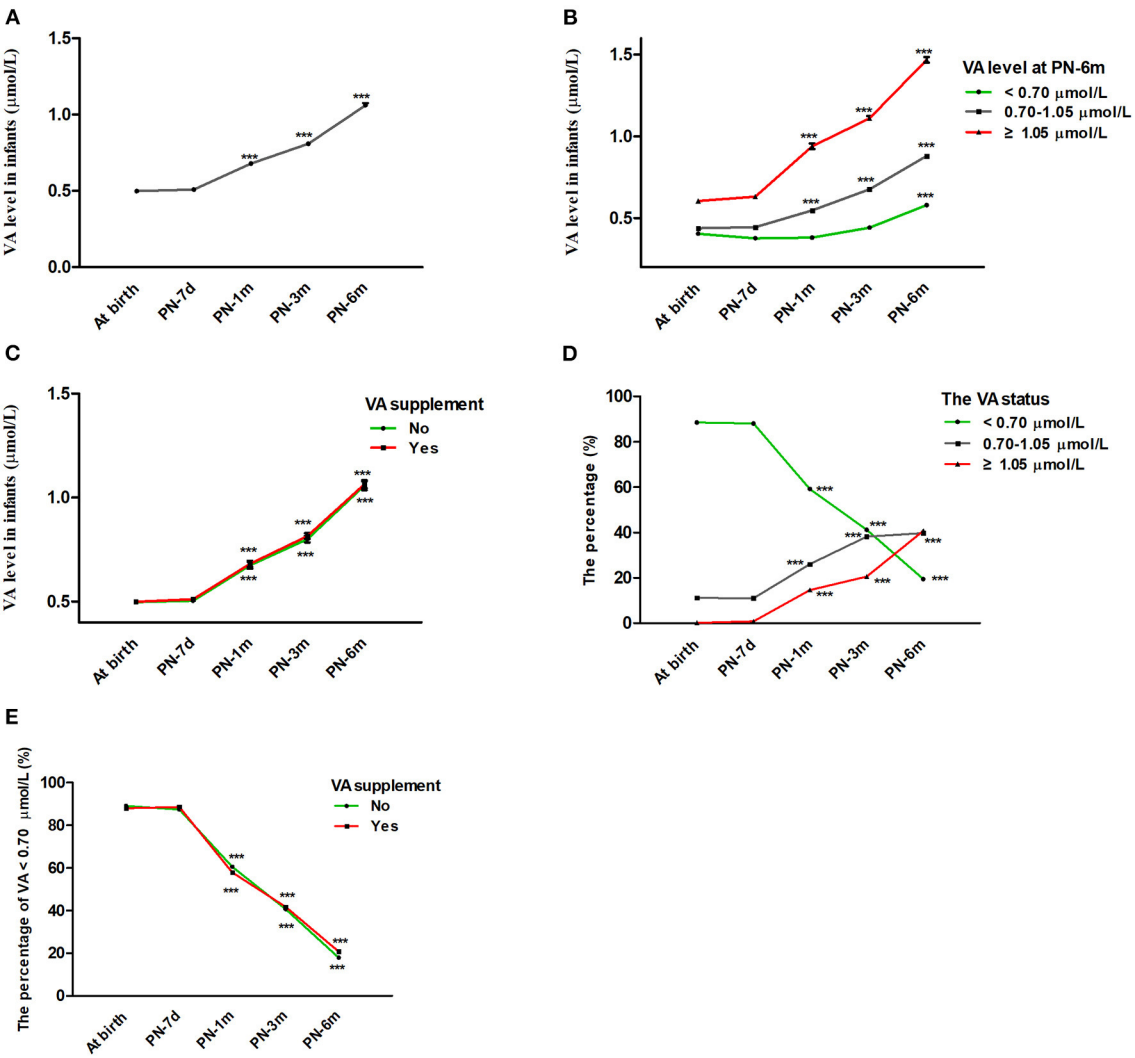
Changes in infant VA status throughout the first 6 months of life. **(A)** Changes in VA levels in infants with age from birth to 6 months. **(B)** Change in VA levels in infants with age after stratification by VA levels at 6 months (VA <0.70, 0.70–1.05, ≥1.05 μmol/L). **(C)** Change in VA levels in infants with age after stratification by vitamin A supplementation. **(D)** Changes in VA distribution (VA <0.70, 0.70–1.05, ≥1.05 μmol/L) in infants with age. **(E)** Percentage of infants with VA <0.70 μmol/L vs. age after stratification by vitamin A supplementation. The values are the mean ± SEM **(A–C)** or percentage **(D,E)**. Generalized estimating equations with Bonferroni *post hoc* test. *: significant difference with the last visit (***p < 0.001). VA, vitamin A; PN-7d, postnatal day 7; PN-1m, postnatal month 1; PN-3m, postnatal month 3; PN-6m, postnatal month 6.

The average serum VA concentrations of the infants at birth and at 7 days, 1 month, 3 months, and 6 months postnatally were 0.499 ± 0.146, 0.508 ± 0.165, 0.678 ± 0.325, 0.808 ± 0.314, and 1.061 ± 0.414 μmol/L, respectively (Table 1). According to generalized estimating equation analysis, the VA concentration of the infants increased significantly with age overall after birth (*P* < 0.05) (Figure 2A). Since the existing VA level classification criteria were designed for children aged 6–70 months (6, 15), it was more appropriate to divide infants into three groups of VA status (VAD, VA <0.70 μmol/L; marginal VAD, VA 0.70–1.05 μmol/L; adequate VA, VA ≥1.05 μmol/L) based on the VA level at 6 months (Figure 2B). The VA levels increased significantly with age in the different VA status groups (*P* < 0.05). Similar trends were also observed when analyzed after stratification by VA supplementation (*P* < 0.05) (Figure 2C).

Correspondingly, the percentage of infants with VA levels ≥ 1.05 μmol/L and 0.70–1.05 μmol/L rose from 0.2 to 40.8% and from 11.2 to 39.8%, respectively, while the percentage of infants with VA <0.70 μmol/L decreased from 88.6 to 19.5% from birth to 6 months, respectively (Table 1 and Figure 2D). The proportion of infants with VA levels <0.70 μmol/L also dropped significantly with age after stratification by VA supplementation (*P* < 0.05) (Figure 2E). In addition, during follow-up, no infant demonstrated clinical VAD conditions, such as night blindness, conjunctival xerosis or Bitot's spots. Altogether, these results suggested that serum VA status increased with age throughout the first 6 months of life regardless of initial VA status or the use of VA supplementation.

### VA Levels at Birth in Different Groups According to VA Levels at 6 Months

Next, we explored which ranges of VA levels at birth can lead to adequate VA levels (VA ≥1.05 μmol/L) at 6 months and which cannot (VA <1.05 μmol/L) to find cutoff points for grouping neonatal VA levels. First, the infants were divided into three groups according to the VA level at 6 months (VAD, VA <0.70 μmol/L; marginal VAD, VA 0.70–1.05 μmol/L; adequate VA, VA ≥1.05 μmol/L) (6, 15). Then, we reviewed the VA level at birth for each group, and the 95% interval of the VA level was analyzed as the VA reference interval at birth for each group. The VA reference interval at birth in the VA ≥1.05 μmol/L group was 0.588–0.620 μmol/L, indicating that infants with VA ≥0.588–0.620 μmol/L at birth gradually improved to adequate VA (VA ≥1.05 μmol/L) at 6 months (Table 2 and Figure 2B). The VA reference interval at birth in the VA 0.7–1.05 μmol/L group was 0.430–0.448 μmol/L and that in the VA <0.7 μmol/L group was 0.392–0.417 μmol/L (Table 2 and Figure 2B). Furthermore, the generalized estimating equations showed that in the <0.70 μmol/L group, those who took VA supplements showed a significantly higher VA level over age, before or after adjustment for feeding pattern and sex (both *P* < 0.05) (Table 2). Therefore, we further analyzed the VA reference interval at birth in the <0.70 μmol/L group stratified by VA supplementation. The VA reference interval at birth in the VA <0.70 μmol/L group with VA supplementation was 0.395–0.426 μmol/L and in the VA <0.70 μmol/L group without VA supplementation was 0.376–0.418 μmol/L. This result indicated that infants with VA 0.430–0.448 μmol/L and ≤ 0.376–0.426 μmol/L at birth still had marginal VAD (VA 0.70–1.05 μmol/L) and VAD (VA <0.70 μmol/L), respectively, at 6 months, despite their VA levels increasing slowly over age. Utilizing 0.430 and 0.588 μmol/L as the cutoff points, neonates at birth can be divided into three groups with low, medium and high VA levels (VA <0.430 μmol/L, 0.430–0.588 μmol/L and ≥0.588 μmol/L, respectively).

**Table 2:** The VA levels at birth in different groups according to VA levels at PN-6m.

		**Crude model**^**a**^		**Adjusted model**^**b**^		**VA level^**#**^ at birth**
		**β (95% CI)**	***P***	**β (95% CI)**	***P***	**2.5th−97.5th percentile (*n*)**
VA <0.70 μmol/L at PN-6m						0.392–0.417 (160)
VA supplement	No	−0.079 (−0.106–0.051)	***P*** **<** **0.001**	−0.077 (−0.105–0.049)	***P*** **<** **0.001**	0.376–0.418 (69)
	Yes	ref		ref		0.395–0.426 (91)
VA 0.70–1.05 μmol/L at PN-6m						0.430–0.448 (327)
VA supplement	No	−0.008 (−0.032–0.016)	0.526	−0.007 (−0.031–0.017)	0.553	
	Yes	ref		ref		
VA ≥1.05 μmol/L at PN-6m						0.588–0.620 (335)
VA supplement	No	0.012 (−0.042–0.066)	0.659	0.000 (−0.054–0.055)	0.99	
	Yes	ref		ref		

a *Generalized estimating equations testing the relationship of VA supplement and VA level in infants over age*.

b *Adjusted for: feeding patterns (Breast feeding vs. Mixed feeding vs. Artificial feeding), sex (Female vs. Male); P-Value for interaction between the vitamin A supplement variable and age in both crude model and adjusted model <0.05*.

# *The VA level is measured in μmol/L. VA, vitamin A; CI, confidence interval. Bold P-values mean significance. PN-6m, postnatal 6 months*.

### Effect of VA Supplementation on Infant Serum VA Level With Age

**Figure 3:**
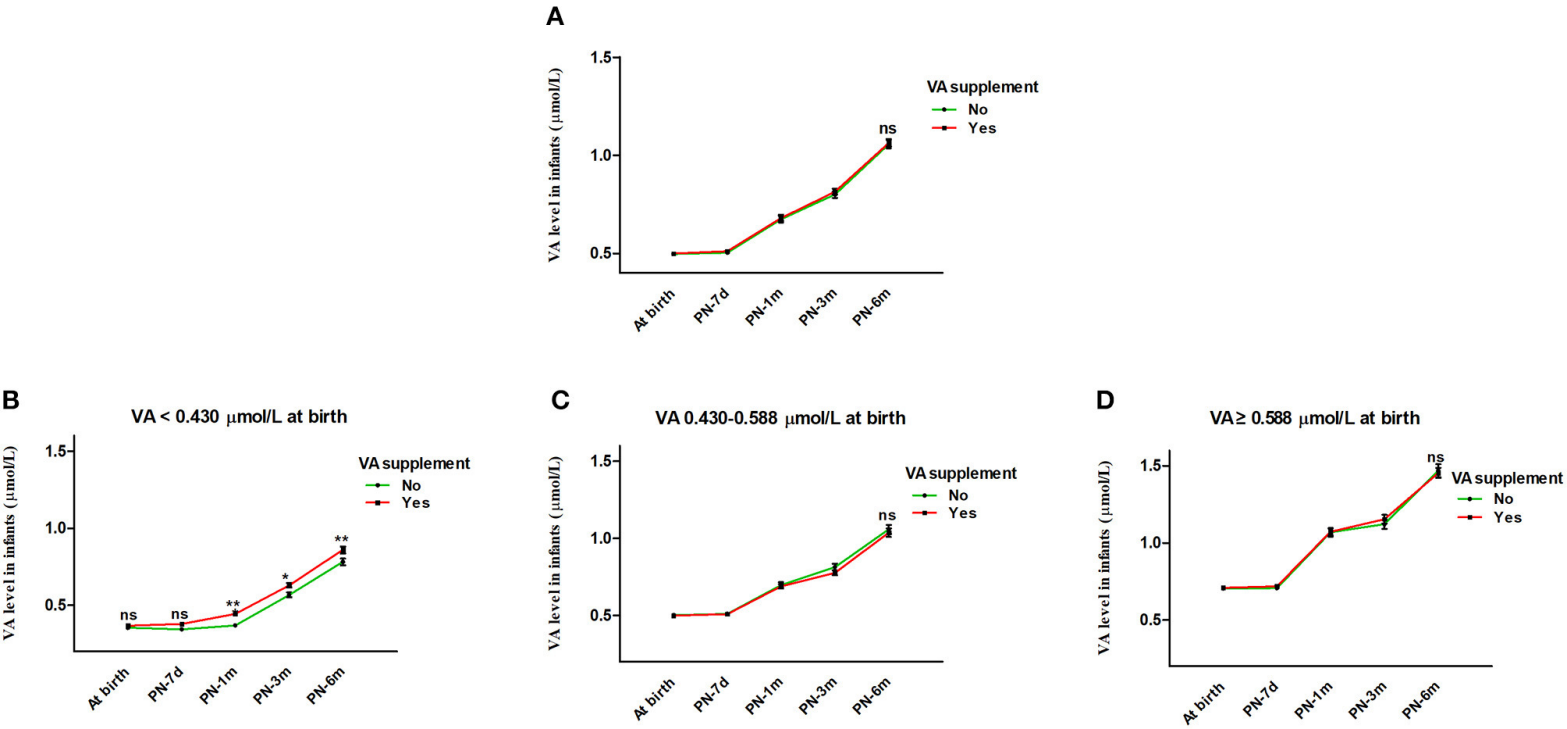
Effect of VA supplementation on infant serum VA level with age. **(A)** Effect of VA supplementation on infant serum VA level with age from birth to 6 months. **(B–D)** Infants were stratified by VA level at birth into VA <0.430 μmol/L, VA 0.430-0.588 μmol/L and VA ≥0.588 μmol/L groups. **(B)** Effect of VA supplementation on infant serum VA level with age in the VA <0.430 μmol/L group. **(C)** Effect of VA supplementation on infant serum VA level with age in the VA 0.430-0.588 μmol/L group. **(D)** Effect of VA supplementation on infant serum VA level with age in the VA ≥0.588 μmol/L group. The values are the mean ± SEM. Generalized estimating equations with Bonferroni *post hoc* test, controlling for the effects of feeding pattern (Breast feeding vs. Mixed feeding vs. Artificial feeding) and sex (Female vs. Male). *Significant difference (*p < 0.05, **p < 0.01) in VA levels between different groups according to VA supplementation at that visit, ns: no significant difference in VA level between different groups according to VA supplementation at that visit **(B)** or for all visits **(A,C,D)**. VA, vitamin A; PN-7d, postnatal day 7; PN-1m, postnatal month 1; PN-3m, postnatal month 3; PN-6m, postnatal month 6.

Generalized estimating equations were then constructed to explore the effect of VA supplementation on VA level over age. There was no significant difference in the VA level between the VA supplementation and non-supplementation groups over age before or after adjustment for feeding patterns and sex (both *P* > 0.05) (Table 3). The curves of age vs. VA level were almost identical between the VA supplementation and non-supplementation groups (Figure 3A).

**Table 3:** Generalized estimating equations testing the relationship of VA supplementation and VA level in infants over age in Chongqing, Southwest China.

	**Crude model**		**Adjusted model**^**a**^	
	**β (95% CI)**	***P***	**β (95% CI)**	***P***
**VA supplement**				
No	−0.031 (−0.068–0.005)	0.094	−0.031 (−0.068–0.006)	0.099
Yes	ref		ref	
**VA** ** <0.430** **μmol/L at birth**				
VA supplement				
No	−0.080 (−0.117–0.044)	***P*** **<** **0.001**	−0.080 (−0.116–0.044)	***P*** ** <0.001**
Yes	ref		ref	
**VA 0.430-0.588** **μmol/L at birth**				
VA supplement				
No	0.006 (−0.038–0.050)	0.801	0.001 (−0.043–0.045)	0.953
Yes	ref		ref	
**VA** **≥0.588** **μmol/L at birth**				
VA supplement				
No	−0.003 (−0.069–0.063)	0.939	−0.004 (−0.070–0.063)	0.917
Yes	ref		ref	

a *Adjusted for: feeding pattern (Breast feeding vs. Mixed feeding vs. Artificial feeding), sex (Female vs. Male); P-Value for interaction between the vitamin A supplementation variable and age in both crude model and adjusted model <0.05*.

*VA, vitamin A; CI, confidence interval. Bold P-values mean significance*.

In addition, we further analyzed the influence of VA supplementation on VA level stratified by VA status at birth. As shown in Table 2, the infants were divided into three groups with low, medium and high VA levels (VA <0.430 μmol/L, 0.430–0.588 μmol/L and ≥0.588 μmol/L). Among the infants with VA levels <0.430 μmol/L at birth, those who took VA supplements showed a significantly higher VA level from 1 month to 6 months before or after adjustment for feeding pattern and sex (both *P* < 0.05) (Table 3 and Figure 3B). VA levels increased by 0.08 μmol/L more in the supplement group than in the non-supplement group. However, among the infants with VA levels of 0.430–0.588 μmol/L or ≥0.588 μmol/L at birth, there was no significant difference in VA level between the VA supplementation and non-supplementation groups over age before or after adjustment for feeding pattern and sex (both *P* > 0.05) (Table 3 and Figures 3C,D). These results indicated that VA supplementation may have an effect on the VA levels of neonates with low VA status at birth (VA <0.430 μmol/L).

## Discussion

According to the WHO (6), a serum VA level <0.70 μmol/L is the diagnostic criterion for VAD in adults and older children aged 6–70 months. In this study, we found very low VA levels (0.499 μmol/L) and a high percentage of infants with VA <0.70 μmol/L (88.6%) among healthy infants at birth. This is consistent with previous results about neonatal VA status from our group and most other research groups (13–17). Previous studies have found that diet, geography, culture, age, preterm birth and infectious disease were important factors affecting vitamin A levels (6, 27). The neonates included in this study were all healthy and born full term (16, 17), indicating that infection and preterm birth were not the determining factors for the high prevalence of VA levels below 0.70 μmol/L (16). However, pregnant women (23.3%) in this study, or pregnant women (12.8%) (17) and older children (13.8%) (18) in other studies from Chongqing, China, were much less likely to have a VA level <0.70 μmol/L than neonates. We also observed that after controlling for the effects of the feeding pattern, VA supplementation and sex, the VA levels of the infants increased with age over the first 6 months of life. This result suggested that rather than dietary environment, geography or culture, young age may be the key factor causing low VA levels in neonates. In addition, the VA levels of most neonates born with a VA level <0.70 μmol/L rose above 0.70 μmol/L at 6 months, even without intensive nutritional intervention. During follow-up, no infant demonstrated clinical VAD conditions, such as night blindness, conjunctival xerosis or Bitot's spots. The vast majority of infants had normal growth indicators, such as weight, length and head circumference, throughout the first 6 months of life. These results suggested that the low VA levels of neonates at birth may be a normal physiological state that then increases with age.

However, it may be inappropriate to use the VAD diagnostic criteria for adults and older children aged 6–70 months (6) to diagnose VAD in neonates, as this could lead to overestimation of the condition in young infants (16). It is not difficult to explain why in places not known for their high prevalences of VAD, the prevalence of neonatal VAD was so high, as reported in our previous study (17). Therefore, it is necessary to explore the true physiological VA levels of infants in the early postnatal period to avoid a misdiagnosis of VAD and even VA supplementation poisoning after birth. In our research, we first found that the VA level in infants with a VA level ≥0.588 μmol/L at birth could rise above 1.05 μmol/L at 6 months old, which is considered adequate VA according to the diagnostic criteria for 6–70 months of age (6). This meant that 0.588 μmol/L may be the normal, minimal physiological VA level at birth for this population, who thus may not need special nutritional intervention, especially with large amounts of VA supplementation, which could cause VA poisoning (20). Similarly, infants with a VA level <0.430 μmol/L at birth still had VAD (retinol <0.70 μmol/L) at 6 months. These infants may thus be at greater risk for VAD and may require greater nutritional attention.

VA supplementation in children aged 6–59 months has been demonstrably associated with a significant reduction in mortality by 23–30% (28–30). The WHO therefore recommend large-scale VA supplementation for children under 5 years of age to improve child survival (22). However, the outcomes for VA supplementation in children younger than 6 months range from no benefit to potentially beneficial or even potentially harmful in previous studies (31–34). There is wide disagreement throughout the world on the appropriate policy for neonatal VA supplementation (19). Whether VA supplementation in the early neonatal period can improve VA status remains inconclusive (21, 22). One randomized controlled trial reported that at 3 months of age, VA supplementation in neonates significantly increased VA levels, but more than half of the infants still had VAD (VA <0.70 μmol/L) (22). Another trial showed that neonatal VA supplementation had little effect on the VA level at 3 months (21). In this study, among infants with VA levels ≥0.588 or 0.588–0.430 μmol/L at birth, there was no significant difference in the VA level between the neonatal VA supplementation and non-supplementation groups at 6 months. However, interestingly, among infants with VA levels <0.430 μmol/L at birth, those who took VA supplements showed a significantly higher VA level. In other words, VA supplementation had different effects on infants with different levels of VA at birth. For those with high levels of birth VA, VA supplementation had little effect because the VA levels in their bodies may have already been in normal balance, while infants at high risk for VAD may be more sensitive to VA supplements. In another report, we also found that neonatal VA supplementation programs may prove most beneficial in areas where VAD is common (19). Altogether, our results suggested that infants with VA above 0.588 μmol/L may indeed have adequate VA levels and may not need VA supplementation. However, infants with VA below 0.430 μmol/L at birth do need VA supplements in early life and may thus benefit from VA supplementation programs. This study simply proposed a new way of thinking about neonatal VA supplementation, hoping to draw more attention to neonatal VA supplementation and nutrition. Additional research is warranted before VA supplementation programs can be developed.

One major limitation of the present study is that this population was sampled from only one representative corner of China. In the future, a large number of multicenter studies are needed. Due to safety and ethical concerns, this study is not a randomized controlled trial, which may have caused some bias.

In conclusion, the low VA levels of neonates at birth may be a normal physiological state that increases with age, suggesting that the VAD criteria for adults and older children (VA <0.70 μmol/L) may overestimate the percentage of neonates with VAD. The VA status of infants with VA ≥0.588 μmol/L at birth gradually increased to adequate VA (VA ≥1.05 μmol/L) at 6 months; these infants thus may not need special nutritional intervention. Infants with VA <0.430 μmol/L at birth still had VAD at 6 months, however, and their VA levels can be significantly increased by early VA supplementation. More multicenter studies are needed to determine a new cutoff point for the diagnosis of neonatal VAD and the administration of nutritional intervention.

## Data Availability Statement

The raw data supporting the conclusions of this article will be made available by the authors, without undue reservation.

## Ethics Statement

The studies involving human participants were reviewed and approved by the Medical Ethic Committee of Children's Hospital affiliated with Chongqing Medical University (022/2014). Written informed consent to participate in this study was provided by the participants' legal guardian/next of kin.

## Author Contributions

TL, JM, and JC designed research. JM provided technical guidance and financial support for the study. HL, QC, LY, and TY conducted research. HL analyzed data and wrote the paper. TL and JM had primary responsibility for final content. All authors read and approved the final manuscript. All authors agreed on the order in which their names were listed in the manuscript.

## Conflict of Interest

The authors declare that the research was conducted in the absence of any commercial or financial relationships that could be construed as a potential conflict of interest.
