# Supplementary material for: Changes in Vitamin A Levels and the Effect of Early Vitamin A Supplementation on Vitamin A Levels in Infants Throughout the First 6 Months of Life: A Prospective Cohort Study in Chongqing, China
Source: Front Public Health. 2021 Apr 27;9:650823. doi: 10.3389/fpubh.2021.650823 (PMC8110732; doi:10.3389/fpubh.2021.650823)
Supplement: Supplementary file 1 [file Table_1.DOCX]

| **Supplementary table 1** The VA^*^ status of mothers during the third trimester of pregnancy | | |
| --- | --- | --- |
| Characteristic | n | Value (Mean ± SD or Percentage) |
| **Maternal total VA intake (****µg RAE/d)^#^** | 1016 | 1033.2±599.9 |
| <530 | 280 | 27.6% |
| ≥530 | 736 | 72.4% |
| **VA level in serum of mothers (µmol/L)^&^** | 1016 | 1.056±0.468 |
| < 0.70 | 237 | 23.3% |
| 0.70-1.05 | 319 | 31.4% |
| ≥ 1.05 | 460 | 45.3% |
| ^*^ VA: Vitamin A.  ^#^ Maternal VA intake was divided into two levels (<530 µg RAE/d, which indicated inadequate VA intake under the Estimated Average Requirement (EAR), and ≥530 µg RAE/d, which was adequate and met the EAR) according to Dietary Reference Intakes (DRIs) for Chinese residents formulated by the Chinese Nutrition Society in 2013.  ^&^ The VA status of pregnant women was classified according to the following categories from WHO: VAD, VA<0.70 µmol/L; marginal VAD, VA≥0.70–1.05 µmol/L; and adequate VA, VA≥1.05 µmol/L. | | |
